# Impact of CMR parameters on clinical outcome after STEMI: data from a large multi-center study

**DOI:** 10.1186/1532-429X-15-S1-O93

**Published:** 2013-01-30

**Authors:** Suzanne de Waha, Ingo Eitel, Georg Fuernau, Philipp Lurz, Jochen Wöhrle, Henning Suenkel, Josefine Meissner, Sebastian Kerber, Bernward Lauer, Matthias Pauschinger, Ralf Birkemeyer, Christoph Axthelm, Rainer Zimmermann, Steffen Desch, Matthias Gutberlet, Gerhard Schuler, Holger Thiele

**Affiliations:** 1 Department of Internal Medicine/Cardiology, University of Leipzig - Heart Center, Leipzig, Germany; 2 University of Ulm, Ulm, Germany; 3 Herz- und Gefäss-Klinik Bad Neustadt, Bad Neustadt, Germany; 4 Zentralklinik Bad Berka, Bad Berka, Germany; 5 Klinikum Nürnberg, Nürnberg, Germany; 6 Schwarzwald-Baar Klinikum Villingen-Schwenningen, Villingen-Schwenningen, Germany; 7 Klinikum Pirna, Pirna, Germany; 8 Klinikum Pforzheim, Pforzheim, Germany; 9 Department of Diagnostic and Interventional Radiology, University of Leipzig - Heart Center, Leipzig, Germany

## Background

Data on the prognostic value of cardiac magnetic resonance imaging (CMR) parameters in patients with ST-elevation myocardial infarction (STEMI) are limited due to analysis of single-center cohorts and small study sample sizes. Aim of the current study was thus to investigate the impact of infarct size, microvascular obstruction (MO) and myocardial salvage index (MSI) on clinical outcome in a large cohort of STEMI patients derived from a multi-center study.

## Methods

STEMI patients (n=795) reperfused by primary percutaneous coronary intervention (PCI) within 12 hours after symptom onset underwent CMR in 8 centers in Germany. CMR was performed at day 1 to 4 after the index event. Infarct size and microvascular obstruction (MO) were measured 15 minutes after gadolinium injection. T2-weighted and contrast-enhanced CMR were used to calculate the MSI. The primary endpoint was defined as a composite of death, non-fatal myocardial reinfarction and congestive heart failure (MACE). Clinical follow-up was conducted after 12 months.

## Results

Infarct size, MO and MSI were significantly associated with MACE in univariable Cox regression analysis (all p<0.01). In multivariable Cox regression analysis including TIMI-risk score, TIMI-flow pre- and post-PCI, ST-segment resolution, left ventricular ejection fraction, as well as infarct size, MO and MSI, infarct size was independently associated with the occurrence of MACE (HR 1.03, 95% CI 1.01-1.04, p=0.02).

## Conclusions

In this largest multi-center cohort of patients with STEMI undergoing CMR reported so far, infarct size as well as MO and MSI were significantly associated with the occurrence of death, non-fatal myocardial reinfarction and congestive heart failure. Infarct size was identified as an independent predictor for adverse clinical outcome after STEMI even after adjustment for traditional outcome markers.

## Funding

None.

